# A holistic approach in herbicide resistance research and management: from resistance detection to sustainable weed control

**DOI:** 10.1038/s41598-020-77649-z

**Published:** 2020-11-26

**Authors:** Chun Liu, Lucy V. Jackson, Sarah-Jane Hutchings, Daniel Tuesca, Raul Moreno, Eddie Mcindoe, Shiv S. Kaundun

**Affiliations:** 1 Syngenta, Herbicide Bioscience, Jealott’s Hill International Research Centre, Bracknell, RG42 6EY UK; 2 Cátedra de Malezas, Facultad de Ciencias Agrarias, Universidad Nacional de Rosario, S2125ZAA Zavalla, Argentina; 3 Syngenta Argentina, Oficina Central, Av. Libertador 1855, Vicente López, B1638BGE Buenos Aires, Argentina

**Keywords:** Agroecology, Ecological modelling, Genetic variation, Plant evolution

## Abstract

Agricultural weeds can adapt rapidly to human activities as exemplified by the evolution of resistance to herbicides. Despite its multi-faceted nature, herbicide resistance has rarely been researched in a holistic manner. A novel approach combining timely resistance confirmation, investigation of resistance mechanisms, alternative control solutions and population modelling was adopted for the sustainable management of the *Amaranthus palmeri* weed in soybean production systems in Argentina. Here, we show that resistance to glyphosate in the studied population from Cordoba province was mainly due to a P106S target-site mutation in the 5-enolpyruvylshikimate 3-phosphate synthase (*EPSPS*) gene, with minor contributions from *EPSPS* gene duplication/overexpression. Alternative herbicides, such as fomesafen, effectively controlled the glyphosate-resistant plants. Model simulations revealed the tendency of a solo herbicidal input to primarily select for a single resistance mechanism and suggested that residual herbicides, alongside chemical diversity, were important for the sustainable use of these herbicides. We also discuss the value of an interdisciplinary approach for improved understanding of evolving weeds.

## Introduction

Weeds are highly diverse organisms capable of evolving traits to survive detrimental stresses. Agricultural fields are one of the most modified ecosystems where weed populations undergo frequent and intensive selection pressure imposed by anthropogenic activities, primarily via the use of herbicides. The large seed production of some weeds, such as *Amaranthus palmeri*, gives rise to the standing genetic variation in field populations^1,2^. Multiple mechanisms could therefore evolve to confer survival against herbicidal treatments. Herbicides applied at high doses usually select for target-site (TS) mutations while low doses usually boost quantitative resistance, including target gene duplication/overexpression and non-target-site resistance (NTSR)^3^. Different weed control practices may select for different resistance mechanisms towards a same herbicide in the same weed species. For instance, resistance to glyphosate in *A. palmeri* could be endowed, either individually or simultaneously, by reduced uptake, *EPSPS* gene amplification, or a P106S target-site mutation^4–6^. The long-term sustainability of weed control practices becomes difficult to predict under laboratory or field settings due to the diverse nature of weeds, the complex resistance mechanism, and the interfering human activities, as well as the interactions among these factors. In this respect, computer-based population models are useful tools for the cost-effective prediction of the evolutionary dynamics of weed populations under different management programs. So far the majority of resistance models have mainly focused on demonstrating principles and been used as educational tools^7^. This is mainly due to the lack of sufficient knowledge about the weed biology and resistance mechanisms, and the difficulty in representing spatio-temporal variations in the models; the former hinders accurate model predictions and the latter hinders field-specific recommendations.

Multiple biological and anthropogenic factors are involved in the evolutionary process of herbicide resistance, and therefore research has often been advanced from various perspectives. Key areas of study include (i) investigation of poor weed control in the field and geographic distribution of resistant populations^8,9^, (ii) confirmation of resistance using glasshouse and laboratory tests^10–12^, (iii) analysis of resistance mechanisms at the genetic and molecular levels^5,13^, (iv) exploration of alternative control methods of resistant weeds^14,15^, and (v) evaluation of long-term sustainability of weed management strategies using population models^16,17^. Most of the studies have focused on a specific aspect and are therefore incomplete. A holistic approach is needed for a more comprehensive understanding and timely management of resistance.

Here, we have combined the different research aspects, from resistance detection to population modelling, and present a case study on the control of the highly damaging *A. palmeri* in soybean agroecosystems in Argentina.

## Results

### Rapid confirmation of resistance to glyphosate

**Figure 1:**
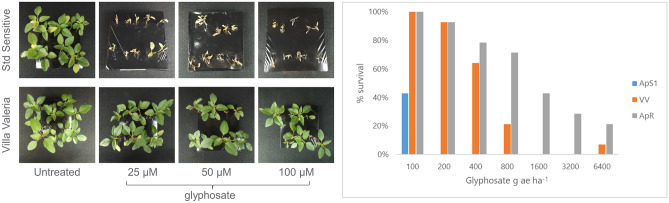
Shift in responses to glyphosate in the *A. palmeri* samples collected in Villa Valeria (VV), Argentina, as compared to the standard sensitive (ApS1) and standard resistant (ApR) populations. Photos from the agar-based RISQ test (left) and dose response from the whole plant pot test (right) are shown.

The standard sensitive *A. palmeri* population (ApS1) was fully controlled at 25 μM in the Syngenta RISQ (Resistance In-Season Quick) test and at 200 g ae ha^−1^ in the whole plant pot test (discriminating rates). Both the standard resistant population (ApR) and the field samples collected from Villa Valeria (VV), Argentina survived the discriminating rates of glyphosate (Fig. 1).

### Mechanism of glyphosate resistance

**Figure 2:**
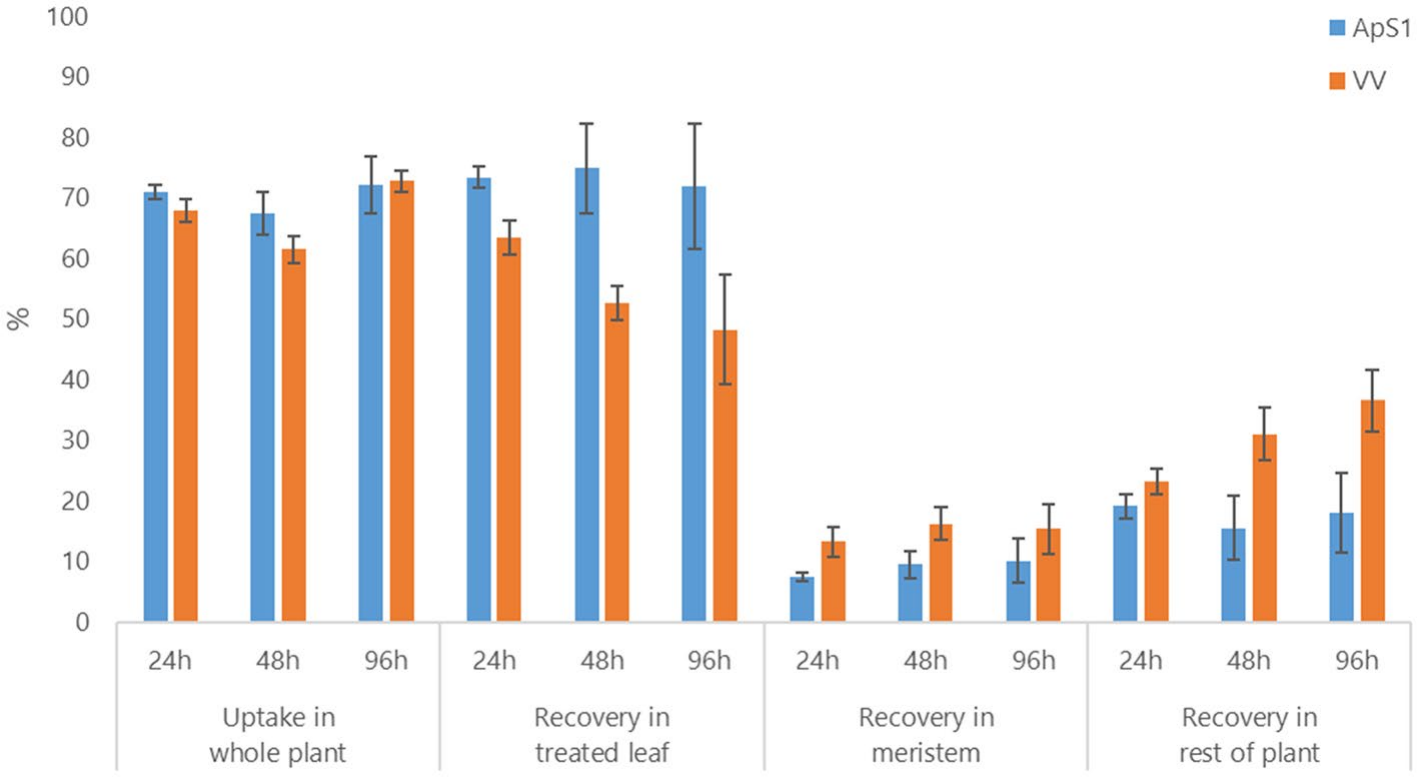
Biokinetics experiment results showing uptake of glyphosate in the whole plant, and radiochemical recovered, expressed as percentage glyphosate absorbed, in different parts of the plant measured at 24, 48 and 96 h after treatment in the ApS1 and VV populations.

There was little evidence to suggest any difference in glyphosate uptake between the VV and ApS1 populations (*p* = 0.2965). Impaired translocation was not observed in the VV population, and contrary to expectations for an impaired translocation concept, the movement to meristem was higher in the VV population than in ApS1 (*p* = 0.0141). Recoveries of glyphosate were over 72% at 96 h after treatment, indicating low metabolic degradation (Fig. 2).

An increase in *EPSPS* gene copy number and expression levels was observed in the VV compared to the ApS2 populations. However, the magnitude of the difference in all cases was relatively small (~ 1.5-fold) (Table 1), as compared to the difference between ApR and ApS1 (~ 9.0-fold) in Kaundun et al.^6^.

**Table 1:** Average *EPSPS* gene copy number (DNA sample) and expression (RNA sample) relative to reference genes *ALS* and *CPS* for the ApS2 and VV populations.

Sample	Gene comparison	ApS2	VV	VV vs. ApS2	
				ratio	*p*-value
DNA	*EPSPS* vs. *CPS*	0.98	1.38	1.41	< 0.0001
DNA	*EPSPS* vs. *ALS*	1.05	1.43	1.36	< 0.0001
RNA	*EPSPS* vs. *CPS*	1.24	2.31	1.86	0.0019
RNA	*EPSPS* vs. *ALS*	0.95	1.28	1.34	0.0054

Sanger sequencing identified the known cytosine (CCA) to thymine (TCA) target-site resistance mutation at *EPSPS* codon 106 in the VV population. The frequencies of the genotypes were 6% for homozygous wild-type PP106, 22% for heterozygous mutant PS106 and 72% for homozygous mutant SS106.

Therefore, resistance to glyphosate in the VV population was endowed by multiple mechanisms, namely, a P106S TS mutation and a low level of *EPSPS* gene duplication/overexpression (quantitative resistance).

### Simulation of evolved glyphosate resistance endowed by different mechanisms

**Figure 3:**
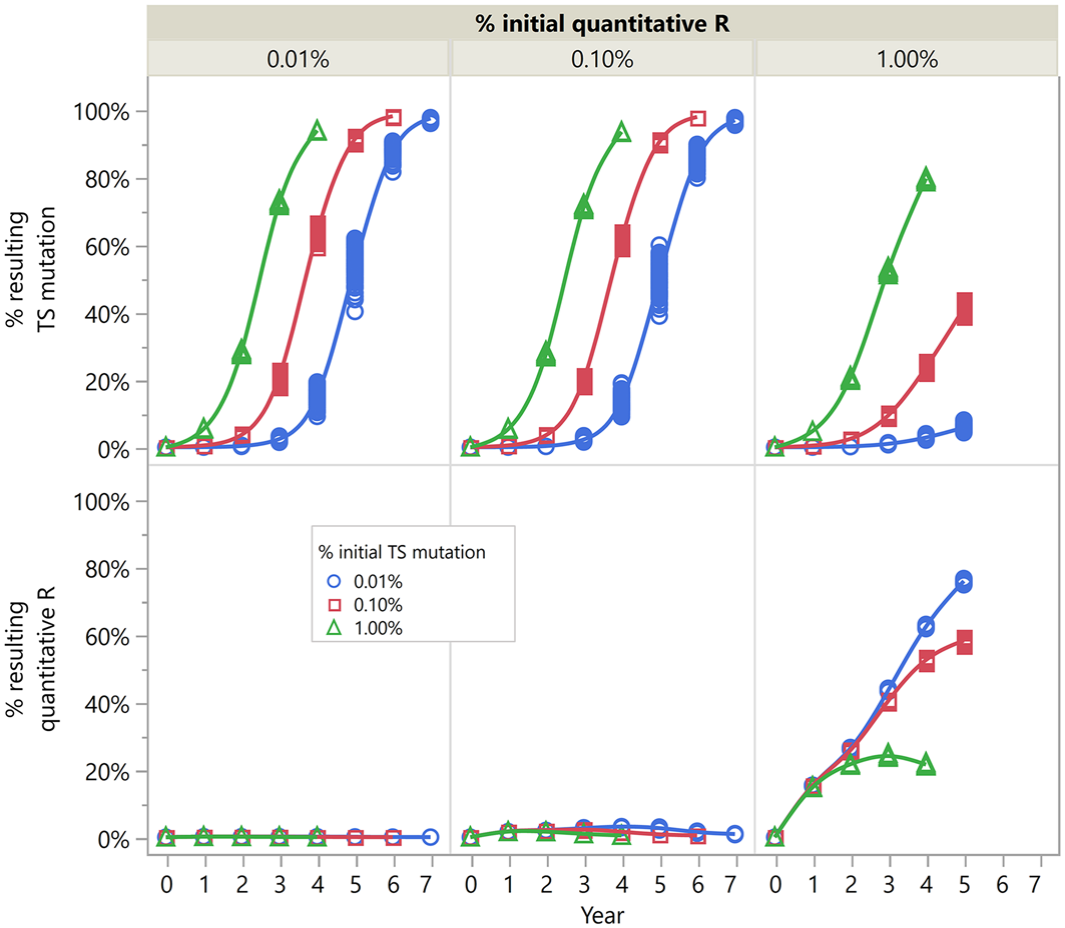
Evolution of glyphosate resistance endowed by target-site (TS) mutation and quantitative resistance respectively, in the surviving plants, with varying initial % resistance (quantitative resistance across columns and TS mutation across different lines). Number of replicates = 100.

The dose responses of PP, PS and SS genotypes (for the *EPSPS* codon 106) in the population model were parameterised based on the VM1 samples (collected from Vicuña Mackenna, Cordoba, Argentina) in Kaundun et al.^6^. This was justified by the similar glyphosate resistance mechanisms in the two populations which were collected near each other (75 km) and had similar previous weed control practices. The model predicted that weed density exceeded control threshold (1 plant m^−2^) in an average of 9.7 years for a pristine population with the two resistance mechanisms implemented simultaneously and glyphosate used as a solo treatment applied twice post-emergence. Evolved TS mutation-based resistance exceeded 20% in an average of 7.4 years, whilst the evolved quantitative resistance was below 0.04% in all of the 100 replicates. For both TS mutation-based and quantitative resistance, the resulting resistance level was positively correlated to the existing number of resistant plants in the population (i.e. initial resistance frequency). However, the level of evolved TS mutation is negatively correlated to the initial % quantitative resistance, and vice versa (Fig. 3). In other words, TS mutation-based resistance and quantitative resistance showed a tendency of being selected in opposite manners. When the population underwent a selection posed by a single herbicide, in this case, glyphosate, a single resistance mechanism would be selected primarily, instead of multiple mechanisms being selected simultaneously. This is because a high initial proportion of individuals with one of the mechanisms would mean the absolute majority of the population that survived the herbicide application carry this particular mechanism (e.g. P106S TS mutation in this case), among which the statistical distribution of the other mechanism (quantitative glyphosate resistance in this case) is nevertheless unskewed, and hence unselected for. This conclusion only holds when a single herbicide is involved. The resistance mechanism to be selected will depend on the relative initial frequency of the candidate mechanisms.

### Alternative herbicides

All alternative herbicides tested, including protoporphyrinogen oxidase (PPO) inhibitors lactofen and fomesafen, and residual herbicides *S*-metolachlor and metribuzin controlled the glyphosate-resistant VV population with 100% efficacy at field application rates.

### Sustainability of PPO-based herbicides

**Figure 4:**
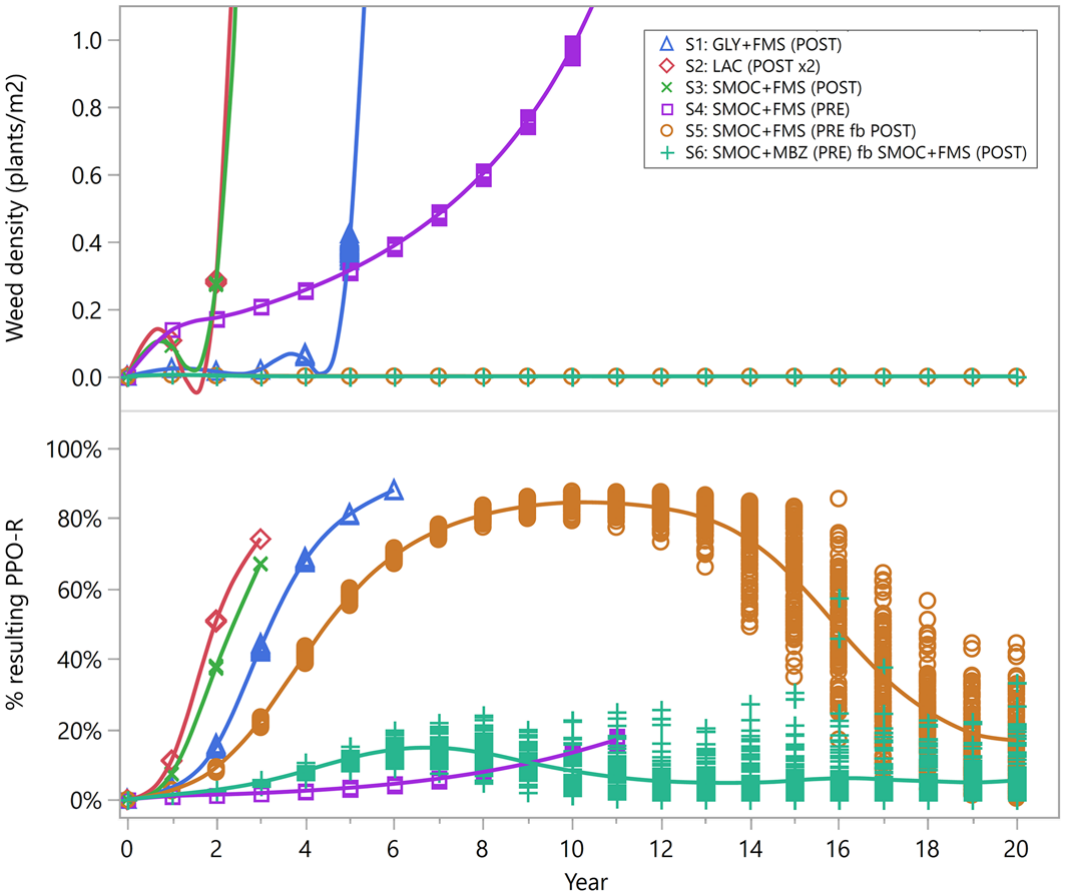
Simulated time series of weed density (upper panel) and % PPO resistance in the soil seedbank (lower panel) under different herbicide programs (S1–S6) in a partially PPO-resistant population (initial proportion of PPO resistance = 10^−2^). Number of replicates in each scenario = 100. *GLY* glyphosate, *FMS* fomesafen, *LAC* lactofen, *SMOC S*-metolachlor, *MBZ* metribuzin, *PRE* pre-emergence application, *POST* post-emergence application, *fb* followed by. For more detail of the scenarios, see Table 2.

The predicted outcome of weed control was affected by both the % exposure to herbicides and the evolution of PPO resistance for populations where glyphosate resistance was commonplace and weed control relied predominantly on PPO-based herbicides. The % exposure is a result of % weed emergence vs. herbicide application timing. A glyphosate + fomesafen mixture at a high application rate, and hence longer residual activity from fomesafen (Table 2), provided sufficient herbicide exposure. However, evolved glyphosate resistance together with partial PPO resistance (10^−2^) led to weed control failure within six years (Fig. 4, S1). Similarly, lactofen, which does not have residual activity, but sequentially applied twice post-emergence, also ensured adequate level of exposure. Using a single herbicide mode of action, however, bore high risk of resistance evolution and led to control failure within three years in partially PPO-resistant populations (S2). In contrast, in programs S3 and S4, *S*-metolachlor + fomesafen was used only once, and so did not control all cohorts of the season. Program S4 was slightly better than S3 because when the mixture of *S*-metolachlor + fomesafen was applied pre-emergence, weed control was from the residual activity of both herbicides. When the mixture was applied post-emergence, the control of early-emerging cohorts relied solely on fomesafen, resulting in high selection pressure. Applying this herbicide mixture both pre-emergence and post-emergence ensured better weed control for a longer period of time (S5) and lower resistance risk than the one-time post-emergence only program (S3). As a further improvement, program S6 incorporated more chemical diversity by replacing fomesafen with metribuzin, a different herbicide mode of action, in the pre-emergence application. In partially PPO-resistant fields (e.g. 10^–2^), S6 effectively remedied PPO resistance evolution from early onset and to a lower level, as compared to S5.

**Table 2:** Detail of the herbicide scenarios tested in the model.

Scenario(s)	Herbicide(s)	Application rate (g ae ha^−1^ or g ai ha^−1^)	Application time	Duration of residual activity (weeks, full/reduced)	POST efficacy (%)
S0	glyphosate	800	POST	N.A	PP: 98.9; PS: 60; SS: 32.2
S1	glyphosate	1550	POST	N.A	PP: 99.9; PS: 87.5; SS: 68.9
	fomesafen	319.5	POST	3.5/5.5	96
S2	lactofen	120	POST	N.A	99
S3, S5 and S6	*S*-metolachlor	1295	POST	3/4	N.A
	fomesafen	285	POST	3/5	95
S4 and S5	*S*-metolachlor	1554	PRE	3.5/4.5	N.A
	fomesafen	342	PRE	3.5/5.5	N.A
S6	*S*-metolachlor	1572.5	PRE	3.5/4.5	N.A
	metribuzin	372.5	PRE	2.5/3.5	N.A

## Discussion

Our holistic study reveals the cause of resistance to glyphosate in the *A. palmeri* population from Argentina, confirms the effectiveness of alternative PPO-based herbicides, and suggests sustainable ways of using the PPO herbicides so that their longevity is maximised. Resistance to glyphosate in this population was due to a major contribution of P106S TS mutation and a minor contribution of *EPSPS* gene duplication/overexpression. This result was different to what is commonly found in the glyphosate-resistant *A. palmeri* populations in the USA which are predominantly characterised by gene duplication/overexpression^5,18,19^. Kaundun et al. tested 115 *A. palmeri* populations from the Midwestern USA, and none of them contained the P106S mutation^6^. This reflects the use of different weed control practices in Argentina and the USA, although recently, the P106S mutation was also found in *Conyza canadensis* in the USA^20^. This may be due to sequential resistance evolution in the herbicide mixture partners. As simulated in the glyphosate-only scenario (Fig. 3), a single herbicidal input was likely to select for a single resistance mechanism, either TS mutation-based or a quantitative trait. This explained the weak contribution of *EPSPS* gene duplication/overexpression in the VV population, whereas P106S TS mutation played the predominant role. In another population of *A. palmeri* from Cordoba province, resistance to glyphosate was exclusively endowed by NTSR of reduced absorption and impaired translocation^21^. Examples in other weed species include a glyphosate-resistant *Poa annua* population from the USA [resistance index (RI) = 17.9], where a sevenfold *EPSPS* gene copy number and a weak P106L mutation coexist, with dominant contribution from gene duplication^22^; and more recently, a glyphosate-resistant *Eleusine indica* population from China (RI = 13.4), where a P106A mutation and a low level of gene duplication/overexpression coexist, with dominant contribution from the TS mutation^23^. In some occasions of resistance to herbicides of other modes of action, such as diclofop-methyl (acetyl-CoA carboxylase inhibitor) and metribuzin (photosystem II inhibitor), TS mutation and NTSR mechanisms had concurrently strong contributions^24,25^. These are likely due to selection pressure from multiple herbicides with the same or different modes of action used in the cropping systems, as opposed to the rather uniform use of solo glyphosate in Argentina. In addition to the high dose vs. low dose regimes^3^, the model showed that initial frequencies of the resistance mechanisms are paramount. It is likely that in the VV population, before *A. palmeri* was subject to herbicidal treatments, the naturally existing P106S mutation was more frequent than the *EPSPS* gene amplification. The P106S mutation was also found in the native *A. quitensis* in the form of a triple *EPSPS* mutation (TAP‐IVS: T102I, A103V and P106S), causing an extremely high level of glyphosate resistance (RI > 300)^26^. Conversely, gene amplification was believed to come from the USA^27^, which could be rare and primarily diluted by the higher frequency of TS mutations. Nonetheless, recent discovery of the extra chromosomal circular DNA-based gene amplification as well as other potentially new mechanisms, such as mobile genetic element insertion, may give rise to higher genome plasticity and more rapid adaptive evolution of the quantitative traits^28,29^. The modelling indications as well as real-life examples provided some different perspective to the appeal that herbicide discovery should aim for multi-target inhibitors^30^, which may not be ubiquitously necessary, considering the common weed control practices in some specific cropping systems. Prior to this work, a population model of a similar weed species, *A. tuberculatus* was built*,* assuming glyphosate resistance as a quantitative trait^31^ and the pace of resistance evolution was faster than the current case of *A. palmeri* in Argentina. The different resistance mechanisms in this Argentine population, together with the insights from the segregation and dose responses of PP, PS and SS genotypes^6^, indicated that it is not always feasible to borrow information from similar or even the same species from other geographic locations for model parameterisation. At the population level, genetic diversity within a weed species could lead to differentiated evolutionary responses to herbicidal as well as agronomic weed control methods^32,33^; at the community level, species diversity could lead to flora changes and succession of dominant weed species^34^. The complex nature of agroecosystems and variations in anthropogenic practices make it difficult and risky to recommend generalised weed management strategies^2,35^. Future weed management programs would benefit from a more localised design, with the help of quick resistance detection methods, molecular assays and predictive modelling, as demonstrated by the holistic approach in this study.

As an alternative to glyphosate, the PPO herbicides effectively controlled the VV population. However, it is important to note that resistance to PPO herbicides has already been documented in *Amaranthus* spp. in the USA and in *Euphorbia heterophylla* and *Conyza sumatrensis* in Brazil^36^, and if not used properly, their sustainability will be at stake (Fig. 4, S1–S3). Model simulations indicated that chemical diversity and the use of residual herbicides were key to the sustainable use of PPO herbicides. With the starting population being partially resistant (initial PPO resistance = 10^−2^), the large variation of resulting PPO resistance in the most effective programs (S5 and S6) warned that resistance could continue to build up even though it may not have been noticeable at the weed density level. If growers switch to a less effective program or a suboptimal dose, the contained high resistance frequency within the seedbank is likely to increase rapidly. Generally, evolutionary processes happen over a much longer time scale than ecological processes^37^. However, the large population size and strong anthropogenic selection pressure embrace contemporary evolution in weed populations at an ecological timescale, which makes it ever more important to detect and take science-based actions at early onset of resistance. To conquer the evolving weeds and weed control challenges, new technologies and concepts, such as weed seed harvest, precision application, artificial intelligence and gene drive systems have attracted growing attention^38–41^. Meanwhile, another level of innovation stems from making use of what is already available in the toolbox and combining the strengths of multiple tools, as demonstrated by this study. Similarly, interdisciplinary research has been proposed to address several ecological issues, such as climate change, fishery management, forestry retention, soil health and disease emergence^42–46^. However, few comprehensive examples exist in practice, for it requires a skilful balance between inputs from different and sometimes conflicting perspectives. The majority of collaborations have been conducted in the form of workshops or training consortiums^46,47^. We have hereby demonstrated a mini version of an interdisciplinary study achieved within a functional research team. Resistance detection and mechanism investigation informed the parameterisation of the population model. Meanwhile, the model set the frame of the study system and guided each piece of the puzzle to be filled by the relevant upstream studies. It has brought more ecological realism and evolutionary relevance into weed science experiments. As Cousens pointed out recently, statistically non-significant results do not necessarily designate no effect in biological sense^48^. From an evolutionary perspective, several non-significant differences in individual measures may jointly lead to a significant difference at the population level, and the effect is likely to be further signified through iterating generations. Population models, by integrating the segmented information, can help capture the subtle but biologically meaningful differences and translate them into long-term consequences. Additionally, the model selection approach^49^ enabled a feedback loop from dry to wet experiments and filtered unrealistic scenarios, which aided the prioritisation of laboratory work. Finally, the interactions with the knowledge base inspired the model structure to be designed flexibly to allow the incorporation of novel technologies into the system. Further expansion of the holistic approach may include the integration of environmental science, economics, science communication as well as digital tools.

## Methods

### Plant material and growth conditions

The *A. palmeri* samples were collected from a soybean field in Villa Valeria, Cordoba province in Argentina. The field was under soybean monoculture and treated with continuous glyphosate applications for five years prior to seed collection. A standard sensitive (ApS1) population purchased from Azlin Seed Service (USA), a second standard sensitive (ApS2) population and a resistant (ApR) population both sourced from Georgia, USA, were used in glasshouse, molecular and biokinetic experiments. The ApR sample was characterised by *EPSPS* gene duplication/overexpression (ninefold relative to ApS1)^6^. Seeds were sown into trays containing equal portions of compost and peat and maintained in controlled glasshouse conditions of 24/18 °C day/night temperatures and 65% relative humidity. Seedlings at the 1–3 leaf stage were transplanted onto agar for the Resistance In-Season Quick (RISQ) test^10^. Two-centimetre-tall seedlings were transplanted into 75-mm diameter pots and irrigated and fertilised as necessary.

### Glyphosate resistance confirmation tests

The agar-based RISQ test plates contained 0, 25, 50 and 100 µM of glyphosate respectively. Each plate had 10 transplanted *A. palmeri* seedlings and was kept in the glasshouse. Survivorship was assessed 7 to 12 days after transplanting, based on observation of new root and leaf development. In the whole plant pot test, each treatment had 14 individually potted *A. palmeri*. When the plants reached 8 cm in height, they were sprayed with 0, 100, 200, 400, 800, 1600, 3200 and 6400 g ae ha^−1^ of glyphosate. The pots were randomised and maintained in the same glasshouse conditions as mentioned above. Survivorship was assessed 21 days after transplanting.

### Mechanism of resistance to glyphosate

Experiments followed the protocols detailed in Kaundun et al.^6^ and are briefly summarised below.

To test the uptake and translocation of glyphosate, plants from the VV and ApS1 populations were treated with [phosphonomethylene]-^14^C glyphosate at the 4-leaf stage. Each plant received 20 μg glyphosate, with 5 kBq radioactivity. Plants were sampled at 0, 24, 48 and 96 h after treatment, with four replicates for each time point. Treated leaves were painted with cellulose acetate, left to dry, and then dissolved in 1 ml acetone. Radioactivity in the solution was measured by liquid scintillation counting (LSC) using a Perkin Elmer Tricarb 2900TR (Perkin Elmer, Waltham, Ma, USA). Treated plants were freeze-dried and separated into treated leaf, meristem and rest of plant which were then individually combusted in a Harvey OX 500 Biological Oxidiser with attached Zinsser robot (R. J. Harvey Instruments, Frankfurt, Germany). Radioactivity was measured by LSC. Uptake was calculated as percentage of glyphosate applied while translocation to the meristem and rest of plants was evaluated as percentage of total glyphosate absorbed.

Sixteen untreated plants from the VV populations were analysed with quantitative real-time PCR (qPCR) for *EPSPS* gene duplication and overexpression. The ApS2 population were used as sensitive control. DNA was extracted from fresh leaf tissues of each plant with a KINGFISHER Flex Purification System (ThermoFisher Scientific, Leicestershire, UK) and a Wizard Magnetic 96 DNA Plant System (Promega, WI, USA), and used in the gene duplication test. RNA was extracted with the RNeasy Plant Mini Kit (Qiagen, Manchester, UK) and employed in the gene expression analysis. The RNA samples were cleaned with a DNAse at 37 °C for 2 h, after which the enzyme was inactivated at 75 °C for 5 min. Corresponding cDNAs were synthesised using High-Capacity cDNA Reverse Transcription Kit (ThermoFisher Scientific, Leicestershire, UK). Acetolactate synthase (*ALS*) and carbamoyl phosphate synthetase (*CPS*) were used as reference genes in the tests. The primers consisted of *ALS*-forward 5′-TTCCTCGACATGAACAAGGTG-3′, *ALS*-reverse 5′-CCAACGCGTCCAGTAGCA-3′ and *ALS-*probe 5′-TTTTCGCTGCTGAAGGCTACGCTC-3′; *CPS*-forward 5′-TGCGGCAATTTTAAGAGCAT-3′, *CPS*-reverse 5′-GATGAGCTGAAGATTGAACAACCT-3′ and *CPS*-probe 5′-AGCTTCACTCCTAGCGATGCCTCCC-3′; *EPSPS*-forward 5′-GTCTAAAGCAACTTGGTTCAGATGT-3′, *EPSPS*-reverse 5′-CCCTGGAAGGCCTCCTTT-3′ and *EPSPS*-probe 5′-TGTTTTCTTGGCACAAATTGCCCTCC-3′. Reactions contained 1× Sigma JumpStart Taq ReadyMix, 300 nM of forward and reverse primers, 100 nM Probe, 3 µl of DNA or cDNA, and distilled H_2_O to 10 µl. Two replicates were used in the tests. All samples were placed in a completely randomised design on the 384-well plate and analysed in a QuantStudio 7 Flex Real-Time PCR System (ThermoFisher Scientific, Leicestershire, USA) using the following settings: 5 min at 95 °C, 40 cycles of 5 s at 95 °C, and 30 s at 60 °C.

Eighteen untreated plants from the VV population were sequenced for known *EPSPS* gene mutations around codon positions 102 and 106. DNA was extracted as described above. Forward (5′ATGTTGGACGCTCTCAGAACTCTTGGT3′) and reverse (5′TGAATTTCCTCCAGCAACGGCAA3′) primers were used to amplify a 195 bp fragment. The reaction was prepared in 25 µl units, each containing 10–50 ng DNA and 20 pmol primers. PCR program was set as follows: 5 min at 95 °C, 30 cycles of 30 s at 95 °C, 30 s at 60 °C, 1 min at 72 °C, and a final 10 min at 72 °C, using a Master Cycle Gradient Thermocycler Model 96 (Eppendorf, UK). Direct Sanger sequencing (Genewiz LLC, USA) was carried out on the PCR product and sequencing reads were aligned and compared using the Seqman software (DNASTAR Lasergene 10, DNASTAR, USA).

### Statistical analysis

The gene copy number (DNA measures) and expression (cDNA measures) from the qPCR experiment were analysed separately. Analysis of variance was performed using SAS version 9.4 (SAS Institute Inc., Cary, NC, USA), by fitting:

1 $${\text{y}}_{{{\text{ij}}}} = \, \mu \, + \, \gamma_{{\text{j}}} + \, \varepsilon_{{{\text{ij}}}}$$

where y_ij_ denotes the difference between the average C_T_ for *EPSPS* and the reference genes for plant i of population j, μ denotes the overall true mean, γ_j_ denotes the effect of population j, and ε_ij_ denotes the random error associated with plant i of population j. A t-test was performed to compare the gene duplication and overexpression levels between the populations, with *p*-value < 0.05 indicating a significant difference.

Similarly, biokinetic measures of glyphosate uptake and translocation were tested by factorial analysis of variance:

2 $${\text{y}}_{{{\text{ijk}}}} = \, \mu \, + \, \gamma_{{\text{j}}} + \, \tau_{{\text{k}}} + \, (\gamma \tau )_{{{\text{jk}}}} + \, \varepsilon_{{{\text{ijk}}}}$$

where i represents replicate, j represents population and k represents time point, τ_k_ denotes the true effect for time k, (γτ)_jk_ denotes the population × time interaction. Where there was evidence of a population × time interaction, the difference between populations were analysed separately at each time point. Otherwise, the comparison was made based on average values across time.

### Model and simulation settings

The model structure and algorithms followed the model of Liu et al.^31^. The individual-based model accounted for the annual life cycle and resistance profiles of the *A. palmeri* population. Herbicide applications removed weed plants from the population, the proportion of which was dependent on the overlap between application time and weed emergence, as well as the standard control efficacy of the herbicide (Table 2). Time-series population dynamics and evolution of resistance emerged from the 20-year iterations. The model was implemented in NetLogo 6.0^50^. Parameters that were specifically updated for *A. palmeri* in the VV population include:emergence curve

3 $${y}_{emg}=\frac{2.65}{73.45}{\times \left(\frac{x}{73.45}\right)}^{2.65-1}\times {e}^{-{\left(\frac{x}{73.45}\right)}^{2.65}}$$

seed production per plant

4 $${f}_{i}=-23008\times ln\left({x}_{i}\right)+113122$$

and annual mortality rate per plant

5 $${mort}_{i}=1.13\times {x}_{i}-63.02$$

where x denotes days after the start of the season (DASS). Sigma of the log-normal distribution of glyphosate quantitative resistance was 0.4656.

The model tested seven herbicidal weed control scenarios (Table 2), one with glyphosate-only program (S0, Fig. 3) and the rest with PPO-based herbicides (S1–S6, Fig. 4). Soybean was planted on 10th November (71 DASS). All weed plants that emerged before sowing were assumed to be controlled by pre-plant burndown applications. Pre-emergence treatments were applied on the same day as planting, i.e. 0 days after planting (DAP). In S0, glyphosate was applied twice post-emergence, 20 DAP and 40 DAP, respectively. Plants that emerged before the application dates were exposed to the herbicide treatment. If the individual plant had a resistance phenotype of the quantitative trait that was higher than the application rate, the plant would survive the treatment; otherwise the survivorship followed the dose–response curves of segregated PP, PS and SS populations described in Kaundun et al.^6^ (Table 2). In S1–S6, two PPO-based herbicides, lactofen and fomesafen were tested. Resistance to PPO-herbicides was assumed to be endowed by a target-site mutation with a dominance value of 0.75^51–53^. The exposure to lactofen was similar to that of glyphosate and survivorship was dependent on the genotype, i.e. 100% of the homozygous resistant individuals and 75% of the heterozygous resistant individuals survived. Fomesafen’s effect was implemented in a similar way to lactofen, with additional residual activity which controlled plants that were to emerge after the application date. Other herbicides included *S*-metolachlor and metribuzin, which only had residual activities. Post-emergence treatments in S5 and S6 were applied on 39 DAP and 32 DAP, respectively. The initial level of resistance to glyphosate was varied to represent different field situations, whilst all populations were assumed to be susceptible to *S*-metolachlor and metribuzin based on current knowledge^36^.

## Data Availability

The datasets generated and/or analysed during the current study are available from the corresponding author on reasonable request.
